# Disengagement of HIV-positive pregnant and postpartum women from antiretroviral therapy services: a cohort study

**DOI:** 10.7448/IAS.17.1.19242

**Published:** 2014-10-08

**Authors:** Tamsin Phillips, Elizabeth Thebus, Linda-Gail Bekker, James Mcintyre, Elaine J Abrams, Landon Myer

**Affiliations:** 1 Division of Epidemiology and Biostatistics, School of Public Health and Family Medicine, University of Cape Town, Cape Town, South Africa; 2 Desmond Tutu HIV Centre, Institute of Infectious Diseases & Molecular Medicine, University of Cape Town, Cape Town, South Africa; 3 Anova Health Institute, Johannesburg, South Africa; 4 ICAP, Mailman School of Public Health & College of Physicians & Surgeons, Columbia University, New York, United States

**Keywords:** antiretroviral therapy, pregnancy, postpartum, retention, prevention of mother-to-child transmission (PMTCT), HIV/AIDS, South Africa

## Abstract

**Introduction:**

Recent international guidelines call for expanded access to triple-drug antiretroviral therapy (ART) in HIV-positive women during pregnancy and postpartum. However, high levels of non-adherence and/or disengagement from care may attenuate the benefits of ART for HIV transmission and maternal health. We examined the frequency and predictors of disengagement from care among women initiating ART during pregnancy in Cape Town, South Africa.

**Methods:**

We used routine medical records to follow-up pregnant women initiating ART within prevention of mother-to-child transmission of HIV services in Cape Town, South Africa. Outcomes assessed through six months postpartum were (1) disengagement (no attendance within 56 days of a scheduled visit) and (2) missed visits (returning to care 14–56 days late for a scheduled visit).

**Results:**

A total of 358 women (median age, 28 years; median gestational age, 26 weeks) initiated ART during pregnancy. By six months postpartum, 24% of women (*n*=86) had missed at least one visit and an additional 32% (*n*=115) had disengaged from care; together, 49% of women had either missed a visit or had disengaged by six months postpartum. Disengagement was more than twice as frequent postpartum compared to in the antenatal period (6.2 vs. 2.4 per 100 woman-months, respectively; *p*<0.0001). In a proportional hazards model, later gestational age at initiation (HR: 1.04; 95% CI: 1.00–1.07; *p*=0.030) and being newly diagnosed with HIV (HR: 1.57; 95% CI: 1.07–2.33; *p*=0.022) were significant predictors of disengagement after adjusting for patient age, starting CD4 cell count and site of ART initiation.

**Conclusions:**

These results demonstrate that missed visits and disengagement from care occur frequently, particularly post-delivery, among HIV-positive women initiating ART during pregnancy. Women who are newly diagnosed with HIV may be particularly vulnerable and there is an urgent need for interventions both to promote retention overall, as well as targeting women newly diagnosed with HIV during pregnancy.

## Introduction

Mother-to-child transmission (MTCT) of HIV is the main contributor to new HIV infections in children globally [1]. In 2012, an estimated 260 000 children were newly infected with HIV and 90% of these new infections occurred in Sub-Saharan Africa (SSA) [1]. Maternal viral load is a key determinant of MTCT risk and triple-drug antiretroviral therapy (ART) is highly effective in reducing viral load, in turn reducing MTCT risk and promoting maternal health [2, 3]. Use of ART by HIV-positive pregnant women is currently the standard of care worldwide and the most recent South African national prevention of mother-to-child transmission (PMTCT) guidelines recommend immediate ART initiation in all pregnant and breastfeeding women [4].

The success of ART for both maternal health and PMTCT hinges on initiation timeously during pregnancy and maintenance of high levels of adherence throughout pregnancy and postpartum when breastfeeding. Uptake of ART during pregnancy has improved with the introduction of ART services integrated into antenatal care (ANC) [5–8]. It has also resulted in earlier ART initiation, increasing the time on treatment prior to delivery and further reducing the risk of MTCT [9–13]. However, poor ART adherence and disengagement from care undermine the potential benefits of maternal ART use during pregnancy and/or breastfeeding and confer increased risk of MTCT as well as maternal morbidity and mortality [12, 14, 15].

Several studies have suggested that levels of ART adherence may be lower, and/or disengagement higher, in pregnant and postpartum women compared to non-pregnant adults initiating ART [16–19]. There is a particular concern during the postpartum period [6, 15, 20], when women may encounter multiple barriers to remaining in care and adherent to treatment [15, 21–23]. In light of these concerns, alongside new policies that call for expanded access to lifelong ART in HIV-positive pregnant women [14], there is an urgent need for insights into ART adherence and retention in care among women initiating ART during pregnancy.

This study investigated retention in care, including disengagement and missed ART dispensing visits, following ART initiation by pregnant women in Cape Town, South Africa. The objectives were (1) to describe the frequency of disengagement and missed visits from ART services during pregnancy and up to six months postpartum, and (2) to investigate socio-demographic, clinical and health service factors, in particular the site of ART initiation, that may be predictors of retention in ART services.

## Methods

We conducted a retrospective cohort study of all HIV-positive women who initiated ART during pregnancy between January 2011 and September 2012, after booking for ANC at a large primary-level antenatal clinic in Gugulethu, Cape Town. The population attending this clinic is predominantly black African. Historically, all women found to be ART eligible during ANC were referred for treatment initiation at a general adult ART clinic on the same premises as the ANC facility [9, 13, 24]. In January 2012, integrated nurse-initiated and managed ART services were introduced at the antenatal clinic. After this time, all eligible women started ART in the ANC facility where they continued to receive their ART care along with ANC throughout the antenatal period. Following delivery, women with viral suppression were transferred to general adult ART services after the latter of 20 weeks on treatment or when the infant HIV status had been determined at six weeks of age. Clinical and counselling protocols did not differ significantly between the general ART and the ANC-based ART service, and counselling services were provided by the same team of counsellors during normal working hours across both ART sites.

Throughout the study period, pregnant HIV-positive women who were eligible for ART were started on a combination of tenofovir with lamivudine and either nevirapine or efavirenz [25]. ART eligibility was based on CD4 cell count of ≤350 cells/µl or WHO stage III/IV disease throughout the study period and women attended 1–2 counselling sessions prior to starting ART. At both ART sites, women received a 30-day supply of ART for the first four months on treatment and a 30- or 60-day supply thereafter; follow-up visits were scheduled every 28 or 56 days, accordingly.

### Data collection

Data for this analysis come from a review of routine medical records for all pregnant women registered in the Gugulethu antenatal clinic with documented HIV infection and who initiated ART between January 2011 and September 2012. Demographic, obstetric and clinical characteristics, as well as the ART initiation site and details of visit attendance (including dates of clinic visits, scheduled visits and quantity of ART supplied at each visit) were abstracted from clinic visit and pharmacy records at both the general adult and integrated ART facilities using standardized data abstraction tools. Data were abstracted up to 12 months on ART and missing laboratory and delivery data were obtained from routine laboratory databases.

### Data analysis

Data were analyzed using Stata 12.0 (Stata Corporation, College Station, USA). Descriptive statistics were used to summarise the baseline characteristics of the study population. Bivariate associations were calculated using Chi-squared tests for categorical variables and the Wilcoxon rank-sum test for independent samples of continuous variables. The primary exposure of interest was the site of ART initiation, analyzed as a binary variable denoting general adult ART initiation or ART initiation integrated into ANC. The quantity of ART supplied at each visit was used to determine the next expected visit date and the number of days late were calculated as the difference between the expected ART visit and the date the visit was attended.

The primary outcome of disengagement from care was defined as having 56 days elapsed since the last scheduled visit with no evidence of attendance, treatment collection or transfer out (TFO) [9, 26, 27]. For the purpose of this analysis, women transferred out during the analysis period were censored at the time of transfer. Secondary analyses focused on missed visits as a marker of non-adherence, defined as being more than 14 days late for a visit but returning to care within 56 days; women who had missed a visit may have disengaged from care at a later date. In sensitivity analyses, we examined variability in the time periods used to define disengagement and missed visits, and found that realistic variations in definitions did not influence results appreciably. Antenatal person-time was accrued from ART initiation to the first of: (1) Delivery; (2) TFO or (3) disengagement. For women remaining in care postpartum, person-time accrued from the date of delivery up to the first of: (1) the end of the study period; (2) TFO or (3) disengagement. The date assigned to disengagement was the date of the last expected visit. Kaplan–Meier curves were generated to explore retention in the antenatal and postpartum periods and between the two ART initiation sites. Predictors of disengagement overall, as well as restricted to the antenatal or postpartum periods, were examined using Cox proportional hazards models, with results reported as adjusted hazard ratios (aHR) with 95% confidence intervals (CI). Variables were included in the model if they demonstrated a significant association (*p*<0.05) with the outcome, and/or appeared to confound the associations involving other variables. Time-varying covariates were used to examine the impact of pregnancy status (antenatal versus postpartum) on disengagement.

Ethical approval to abstract data and conduct this analysis was provided by the Human Research Ethics Committee of the University of Cape Town.

## Results

Of 393 women who initiated ART in pregnancy between January 2011 and September 2012, 358 (91%) had patient records available and were included in this analysis. The median age and gestational age at ART initiation were 28 years (IQR 26–33) and 26 weeks (IQR 21–31), respectively (Table 1).

**Table 1:** Demographic, obstetric and clinical characteristics of 358 pregnant women starting ART stratified by site of ART initiation

	General adult ART initiation site *n (%) or median (IQR)*		ANC ART initiation site *n (%) or median (IQR)*		*p*^a^	Total *n (%) or median (IQR)*	
Number of women	142	(40)	216	(60)	–	358	
Total woman-months of observation	1077	(44)	1369	(56)	–	2446	
Demographics							
Age (years)	28	(25–33)	28	(26–33)	0.829	28	(26–33)
Level of education							
Primary	13	(9)	10	(5)	0.100	23	(6)
Secondary/tertiary	128	(90)	199	(92)		327	(91)
Missing	1	(1)	7	(3)		8	(2)
Employment status					0.838		
Unemployed	98	(69)	131	(61)		229	(64)
Employed	42	(30)	59	(27)		101	(28)
Missing	2	(1)	26	(12)		28	(8)
Relationship status					0.014		
Not in a relationship	25	(18)	15	(7)		40	(11)
In a relationship	115	(81)	160	(74)		275	(77)
Missing	2	(1)	41	(19)		43	(12)
HIV history							
Time of HIV diagnosis					0.068		
Diagnosed prior to current pregnancy	67	(47)	79	(37)		146	(41)
Diagnosed in current pregnancy	75	(53)	132	(61)		207	(58)
Missing	0	(0)	5	(2)		5	(1)
Disclosure status					0.021		
Have not disclosed	12	(8)	36	(17)		48	(13)
Have disclosed	129	(91)	174	(81)		303	(85)
Missing	1	(1)	6	(3)		7	(2)
Previous ARV use							
No exposure	44	(31)	114	(53)	<0.001	158	(44)
Previous ART	9	(6)	10	(5)		19	(5)
PMTCT only	88	(62)	87	(40)		175	(49)
Missing	1	(1)	5	(2)		6	(2)
WHO stage at screening							
I/II	120	(85)	194	(90)	0.135	314	(88)
III/IV	22	(15)	22	(10)		44	(12)
CD4 cell count at screening	233	(157–285)	230.5	(157–291)	0.944	233	(157–287)
Obstetric characteristics							
Median parity	1	(1–2)	1	(1–2)	0.195	1	(1–2)
First pregnancy	28	(20)	40	(19)		68	(19)
One child	57	(40)	96	(44)		153	(43)
Two or more children	38	(27)	78	(36)		116	(32)
Missing	19	(13)	2	(1)		21	(6)
Gestational age at initiation	28	(23–32)	25	(20–30)	0.006	26	(21–31)

ARV=antiretroviral; ART=antiretroviral therapy; WHO=World Health Organization.

a Bivariate comparisons using Chi-squared and Wilcoxon rank-sum tests. Missing data excluded.

Most women had at least secondary level education, only 28% of women were employed, and 77% reported being in a relationship. Overall, 207 women (58%) were newly diagnosed as HIV-positive in the current pregnancy and the majority of women reported having disclosed their HIV status to at least one person at the time of ART initiation. The median CD4 cell count at the time of screening for ART eligibility was 233 cells/µl (IQR 157–287) and 88% of women were classified as WHO clinical stage I or II; 19% were in their first pregnancy and the median parity was 1 (IQR 1–2). History of antiretroviral (ARV) exposure was available for 350 women (98%) of whom 45% were ARV naïve, 5% had previously initiated ART and defaulted prior to the index pregnancy and 50% had previous exposure to ARV prophylaxis for PMTCT.

In total, 142 women started ART in the general adult ART service while 216 women initiated ART within the ANC clinic. There was no significant difference in age, parity, education or employment status in the two groups. Among women starting ART in the ANC clinic, 61% had been newly diagnosed with HIV in the current pregnancy compared to 53% of women referred out to start ART (*p*=0.068). Women starting ART in the ANC clinic also tended to have a lower WHO clinical stage at ART initiation (*p*=0.036), although there was no significant difference in screening CD4 cell count between the two groups. Disclosure was common in both groups; however, the proportion of women who had disclosed was significantly higher among women initiating in the adult ART clinic compared to women starting within ANC (*p*=0.021).

Descriptive characteristics of all women stratified by their final retention status in the primary analysis are displayed in Table 2. Of 358 women included in the analysis, 115 women (32%) had disengaged from care while 243 (68%) were either still in care at the ART initiation site or had been TFO before six months postpartum. Demographic characteristics were similar between women retained and those who had disengaged. Among those retained in care, 54% had been newly diagnosed with HIV, compared to 65% new diagnoses among women who had disengaged from care (*p*=0.060). Median gestational age at ART initiation was lower among women who were retained (25 weeks IQR: 20–30), compared to those who had disengaged (28 weeks IQR: 21–32).

**Table 2:** Demographic, obstetric and clinical characteristics of 358 pregnant women starting ART stratified by final retention status

	Lost to follow-up *n (%) or median (IQR)*		Retained *n (%) or median (IQR)*		*p*^a^
Number of women	115	(32)	243	(68)	–
Total woman-months of observation	508	(21)	1938	(79)	–
Integrated ANC/ART initiation site	66	(57)	150	(62)	0.433
Demographics					
Age	28	(24–33)	28	(26–33)	0.576
Level of education					
Primary	6	(5)	17	(7)	0.568
Secondary/tertiary	104	(91)	223	(92)	
Missing	5	(4)	3	(1)	
Employment status					
Unemployed	68	(59)	161	(66)	0.155
Employed	38	(33)	63	(26)	
Missing	9	(8)	19	(8)	
Relationship status					
Not in a relationship	9	(8)	31	(13)	0.277
In a relationship	85	(74)	190	(78)	
Missing	21	(18)	22	(9)	
HIV history					
Time of HIV diagnosis					
Diagnosed prior to current pregnancy	39	(34)	107	(44)	0.060
Diagnosed in current pregnancy	75	(65)	132	(54)	
Missing	1	(1)	4	(2)	
Disclosure status					
Have not disclosed	14	(12)	34	(14)	0.629
Have disclosed	99	(86)	204	(84)	
Missing	2	(2)	5	(2)	
Previous ARV use					
No exposure	51	(44)	107	(44)	0.640
Previous ART	8	(7)	11	(5)	
PMTCT only	55	(48)	120	(49)	
Missing	1	(1)	5	(2)	
WHO stage at screening					
I/II	100	(87)	214	(88)	0.765
III/IV	15	(13)	29	(12)	
CD4 cell count at screening	248	(151–290)	228	(159–285)	0.568
Obstetric characteristics					
Median parity	1	(1–2)	1	(1–2)	0.719
First pregnancy	24	(21)	44	(18)	
One child	44	(38)	109	(45)	
Two or more children	38	(33)	78	(32)	
Missing	9	(8)	12	(5)	
Gestational age at initiation	28	(21–32)	25	(20–30)	0.027

ARV, antiretroviral=ART=antiretroviral therapy; WHO=World Health Organization.

a Bivariate comparisons using Chi-squared and Wilcoxon rank sum tests. Missing data excluded.

Outcomes by ART initiation site are displayed in Table 3. In the primary analysis, 32% of women had disengaged (having 56 days elapsed from the last scheduled visit and no evidence of attendance) and 24% of women experienced a missed visit (returning to care 14–56 days after the scheduled visit date). Overall, 49% of women had either disengaged or had at least one missed visit before six months postpartum. Missed visits were more common in the general ART clinic and in the postpartum period. Rates of disengagement were lower in the antenatal period (2.41 per 100 woman-months) compared to postpartum (6.17 per 100 woman-months). The difference between postpartum and antenatal rates of disengagement was higher among women who started ART in the ANC clinic (rate ratio, 4.09) compared to those starting at a general adult service (rate ratio, 1.42).

**Table 3:** Outcomes stratified by site of ART initiation

	General adult ART initiation site *n (%) or rate (95% CI)*		ANC ART initiation site *n (%) or rate (95% CI)*		*p*	Total *n (%) or rate (95% CI)*	
Overall analysis period (ART initiation to six month postpartum)							
Number of women	142	(40)	216	(60)		358	
Total woman-months of observation	1077	(44)	1369	(56)		2446	
Retained (including TFO)	93	(65)	150	(69)	0.430	243	(68)
Disengaged from care	49	(35)	66	(31)	0.430	115	(32)
Rate of disengagement per 100 woman-months	4.55	(3.44–6.02)	4.82	(3.79–6.14)	0.762	4.70	(3.92–5.64)
One or more missed visit	50	(35)	36	(17)	<0.001	86	(24)
Antenatal period (ART initiation to delivery)							
Number of women	142		216			358	
Total woman-months of observation	340		616			956	
Retained (including TFO)	130	(92)	205	(95)	0.249	335	(94)
Disengaged from care	12	(8)	11	(5)	0.249	23	(6)
Rate of disengagement per 100 woman-months	3.53	(2.00–6.22)	1.79	(0.99–3.23)	0.109	2.41	(1.60–3.62)
One or more missed visit	7	(5)	11	(5)	1.000	18	(5)
Postpartum period (delivery to six months postpartum)							
Number of women	127		205			332	
Total woman-months of observation	737		753			1490	
Retained (including TFO)	90	(71)	150	(73)	0.693	240	(72)
Disengaged from care	37	(29)	55	(27)	0.693	92	(28)
Rate of disengagement per 100 woman-months	5.02	(3.64–6.93)	7.30	(5.61–9.51)	0.077	6.17	(5.03–7.57)
One or more missed visit	43	(34)	27	(13)	<0.001	70	(21)

ART=antiretroviral therapy; TFO=transfer out.

**Figure 1:**
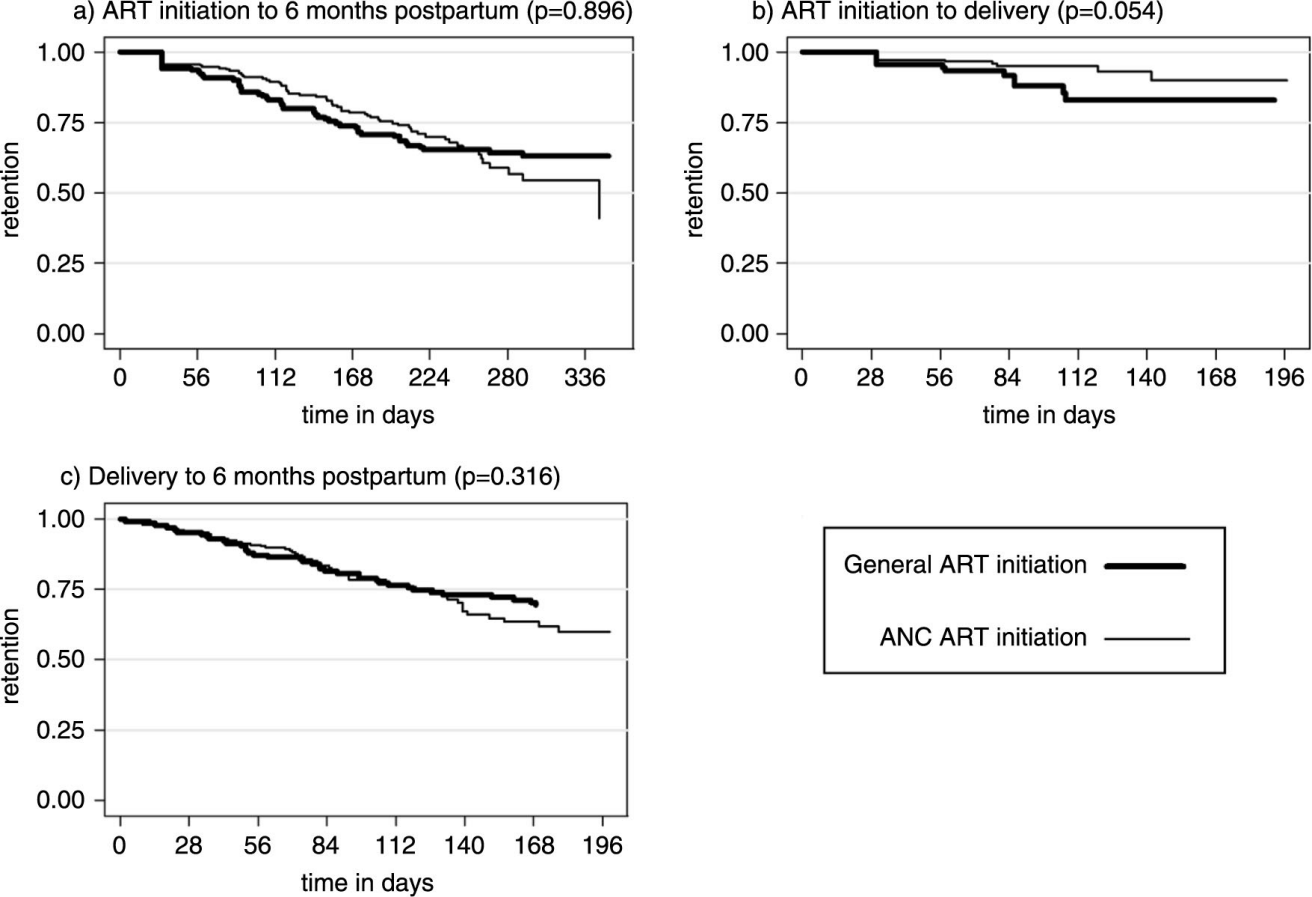
Kaplan–Meier curves of the probability of retention in care for a) ART initiation to six months postpartum, b) ART initiation to delivery and c) delivery to six months postpartum (log rank *p*-values presented).

**Figure 2:**
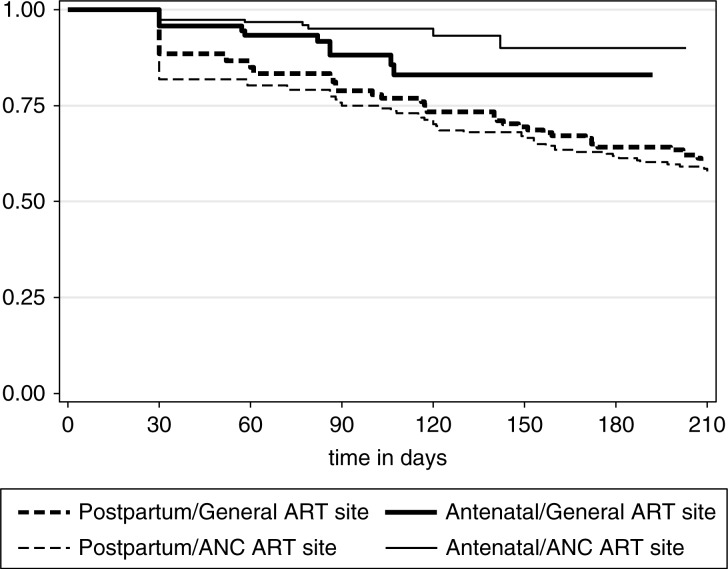
Kaplan–Meier curve of retention during the antenatal and postpartum periods by ART initiation site.

Figure 1 shows disengagement from care by site of treatment initiation overall and separately for the antenatal and postpartum periods. Among women who disengaged from care after delivery, the median time to disengagement was 57 days postpartum (IQR 9–97). Rates of disengagement appeared similar in women who started ART in the general ART clinic compared to those initiated in the ANC overall (Figure 1a) and postpartum (Figure 1c). Disengagement in the antenatal period appeared higher among those who started ART in a general ART clinic compared to among women who initiated ART in the ANC, and this association approached statistical significance (*p*=0.054; Figure 1b). The cumulative probability of disengagement was higher in the postpartum period regardless of the model of ART initiation (Figure 2; *p*=0.0004).

In proportional hazard models (Table 4), the site of ART initiation was not a significant predictor of disengagement overall, antenatally or in the postpartum period. Only having a later gestational age at ART initiation and being newly diagnosed with HIV in the current pregnancy were identified as significant predictors of disengagement overall and after delivery. After adjusting for age, time of HIV diagnosis, CD4 cell count, site of initiation and being in the postpartum period, a one-week increase in gestational age at ART initiation was associated with a 4% increased hazard of having disengaged from care by six months postpartum (aHR: 1.04 CI: 1.00–1.07). Women newly diagnosed with HIV in the current pregnancy had a 57% increased hazard of disengagement overall compared to women with known HIV infection (aHR: 1.57 CI: 1.07–2.33). There was an almost 4-fold increase in the hazard of disengagement postpartum compared to that found in the antenatal period (aHR: 3.93 CI: 1.25–12.31).

**Table 4:** Proportional hazard models predicting disengagement according to participant demographic, obstetric and clinical characteristics in (a) the overall analysis period, and restricted to (b) the antenatal period and (c) the postpartum period

	A) Crude associations			B) Adjusted associations		
		
	HR	(95% CI)	*p*	aHR	(95% CI)	*p*
a) Overall analysis period (ART initiation to six month postpartum) (*n*=353 in multivariate model)						
Older age	0.99	(0.95–1.02)	0.517	0.99	(0.95–1.03)	0.554
Employed	0.80	(0.54–1.19)	0.263			
Diagnosed in current pregnancy	1.56	(1.06–2.30)	0.024	1.57	(1.07–2.33)	0.022
Have not disclosed	0.96	(0.55–1.68)	0.879			
ARV history						
No exposure	1	(ref.)				
Previous ART	1.11	(0.53–2.35)	0.778			
PMTCT only	1.02	(0.70–1.50)	0.912			
WHO clinical stage III/IV	0.97	(0.74–1.27)	0.802			
Increasing screening CD4	1.00	(0.999–1.003)	0.570	1.00	(0.999–1.003)	0.453
Increasing parity	0.98	(0.79–1.20)	0.824			
Increasing gestational age	1.05	(1.02–1.08)	0.001	1.04	(1.00–1.07)	0.030
Integrated ART	1.03	(0.70–1.49)	0.897	1.14	(0.77–1.69)	0.506
Postnatal	2.75	(1.56–4.88)	0.001	3.93	(1.25–12.31)	0.019
b) Antenatal (ART initiation to delivery) (*n*=353 in multivariate model)						
Older age	0.99	(0.91–1.08)	0.866	0.99	(0.91–1.08)	0.860
Employed	2.24	(0.65–7.63)	0.199			
Diagnosed in current pregnancy	1.25	(0.52–2.98)	0.613	1.28	(0.53–3.09)	0.576
Have not disclosed	0.58	(0.14–2.48)	0.462			
WHO clinical stage III/IV	0.59	(0.22–1.62)	0.307			
Increasing screening CD4	1.00	(0.99–1.00)	0.449	1.00	(0.99–1.00)	0.514
Increasing parity	1.04	(0.66–1.64)	0.863			
Increasing gestational age	1.06	(0.98–1.16)	0.145	1.06	(0.97–1.15)	0.193
Integrated ART	0.45	(0.19–1.04)	0.062	0.48	(0.21–1.13)	0.093
c) Postpartum (delivery to six months postpartum) (*n*=327 in multivariate model)						
Older age	0.98	(0.94–1.02)	0.391	0.99	(095–1.03)	0.472
Employed	0.68	(0.44–1.04)	0.077			
Diagnosed in current pregnancy	1.58	(1.03–2.44)	0.038	1.59	(1.03–2.46)	0.038
Have not disclosed	1.08	(0.59–1.99)	0.796			
ARV history						
No exposure	1	(ref.)				
Previous ART	1.25	(0.59–2.67)	0.562			
PMTCT only	0.92	(0.60–1.41)	0.691			
WHO clinical stage III/IV	1.04	(0.78–1.38)	0.788			
Increasing screening CD4	1.00	(0.999–1.004)	0.291	1.00	(0.999–1.004)	0.236
Increasing parity	0.95	(0.74–1.20)	0.651			
Increasing gestational age	1.04	(1.01–1.08)	0.012	1.05	(1.01–1.08)	0.008
Integrated ART	1.24	(0.81–1.89)	0.318	1.31	(0.85–2.02)	0.224

ART=antiretroviral therapy; ARV=antiretroviral; PMTCT=prevention of mother-to-child transmission; WHO=World Health Organization.

## Discussion

This analysis suggests that by six months postpartum, almost half of women had either missed at least one scheduled visit or had disengaged from care after initiating ART during pregnancy and that both disengagement and missed visits appeared substantially more common postpartum compared to before delivery. Overall, 24% of women in this analysis missed at least one scheduled ART dispensing visit and subsequently returned to care, suggesting poor treatment adherence even among those women who remain engaged in care. In the context of pregnancy and PMTCT, these gaps in care and treatment have serious implications for the risk of vertical transmission as well as maternal health.

Missed visits occurred more frequently after delivery but also appeared to be more common among women starting ART in the general ART site. We can hypothesize that there may have been longer waiting times and higher patient volumes in the general ART clinic, presenting an additional burden to mothers on ART. In addition, attending a dedicated ART clinic may be associated with greater HIV-related stigma. These and other possibilities require future research attention.

Although retention-related outcome definitions vary between studies, these findings are broadly consistent with the results of previous publications indicating high levels of non-retention and/or non-adherence, particularly postpartum [15, 26, 28–30]. The rate of disengagement appeared higher in the ANC ART site post-delivery and lower in the ANC ART site prior to delivery, compared to rates among women in the general ART clinic. This supports recent data from Malawi suggesting that integrated ANC ART care may enhance uptake of ART but may have poorer retention outcomes compared to other models of care [31].

Two significant predictors emerged for risk of disengagement in this analysis. First, women newly diagnosed with HIV in pregnancy were more likely to have disengaged from care compared to women who had been diagnosed previously. This echoes previous findings suggesting substantial challenges related to coping with an HIV diagnosis in pregnancy [22, 27, 32]. Women diagnosed with HIV during pregnancy may not be being adequately counselled regarding their HIV status and lifelong treatment. Improving post-test and early ART counselling for women newly diagnosed with HIV may aid ART retention and adherence [33, 34], and will be an important consideration as many countries move towards same-day ART initiation. Studies have found that peer support groups may play a significant role in improving understanding of HIV and PMTCT, and promoting retention and adherence [35–37].

Second, later gestational age at initiation was found to be a predictor of disengagement through six months postpartum. Women initiating ART at later gestational ages have less time in HIV care and possibly less counselling than women who start ART earlier in pregnancy. This may influence retention, although the data are mixed [28]. While the underlying mechanisms require further investigation, it is also possible that late gestation at ART initiation reflects suboptimal health-seeking behaviour more generally [38, 39]. This would suggest that women who seek ANC and/or initiate ART late in pregnancy are a high-risk population that requires special attention throughout the postpartum period of breastfeeding.

Other predictors of disengagement and/or poor adherence identified in existing studies include younger age [16, 26, 28], non-disclosure of HIV status [33, 40, 41] and better baseline disease status [27, 28]. These were not confirmed in this analysis, however the population of women described here did not vary significantly in age and disclosure status, and all women started ART based on CD4≤350 cells/µl. Our ability to assess predictors may have been limited by this lack of variability within covariates. Inadequate knowledge about PMTCT, partner involvement and travel costs have also been identified as barriers to retention and adherence [33, 34, 40, 42], however these variables were not available in this analysis.

We found that women receiving care in the integrated service started ART earlier in gestation. Earlier gestational age at initiation was found to be a predictor of retention in this analysis, however levels of disengagement were similar among women in both ART initiation groups overall. In the antenatal period the frequency of disengagement was lower among women who received integrated ART compared to women who were referred out for ART (1.82 and 3.59 women per 100 women-months, respectively, *p*=0.054). This may be explained by a lower burden of health care visits as well as improved relationships with health care providers in an integrated ART/ANC service [43, 44]. Further research is required to fully explore the impact of timing of ART initiation and integrated service delivery on retention in care as well as adherence both during pregnancy and after delivery.

Linking women to long-term ART care from PMTCT services is known to be a challenge [21, 45]. Although women in this analysis were considered to be retained in care at the time of TFO, it is important to note that women transferred in the early postpartum period may be particularly vulnerable to disengagement. Several studies, including the present analysis, have found that women are at a higher risk of disengagement and/or non-adherence postpartum compared to during pregnancy [15, 26]. This, coupled with the known challenges of linking women to general adult ART care, may be cause for concern [21, 45]. Data on women after they were transferred out of the ANC was not available. There is a clear need for research on postpartum linkage to general ART services to further understand barriers to and enablers of postpartum linkage, as well as to develop interventions to ensure women complete the transition to lifelong adult ART. Further research is also required to establish the optimal timing of postpartum TFO from integrated ART care to ensure women remain adherent and in care during the critical postpartum breastfeeding period.

Several important limitations should be considered with the present findings. The sample size may limit the power of the analysis to detect small associations involving measured covariates. All data were collected from review of routine medical records and not all covariates were routinely measured and recorded in this setting. Related to this, recording of reasons for late or missed visits is not routine and it was not possible to explore reasons for late visit attendance. The retrospective design of this study made it possible to include two sequential cohorts of women initiating ART, first at general adult ART services and then in an integrated antenatal ART service. It is possible that waiting times were longer in the general ART clinic than the integrated clinic due to higher patient volumes however these data were not available. Although there were no notable changes in policy or service delivery models over the review period, without randomization or simultaneous enrolment at the two ART sites, changes in care provision over time could not be controlled for in this analysis.

This analysis is limited to a cohort of women known to have initiated ART during pregnancy; a select group who successfully linked from ANC to ART services. Disengagement prior to ART initiation in pregnancy is known to be high [7, 46] and our estimates may be an underestimation as a result of this selection bias. The rate of disengagement in this analysis may be an overestimate as women who were considered disengaged may have returned to care after the end of the study period or moved to other facilities without formal transfer. Recent studies have suggested that not all patients considered lost to follow-up are truly no longer in care [23, 47, 48]. Results from Zambia suggest that ART patients frequently re-present for care following a period of disengagement, and this possibility warrants further examination in the context of postpartum follow-up of women on ART [49]. In addition, this analysis focused on missed visits and disengagement from care up to six months postpartum but there are other important outcomes, in particular infant HIV infection and maternal retention through breastfeeding for PMTCT, and on-going retention for maternal health which we have not examined.

Despite the limitations of routinely collected medical records, this study took place in a large, representative primary health care facility where visit attendance and pharmacy records were well completed. In turn, these data represent a “real-world” estimate of the timing of disengagement from primary care services in this setting; however, further research into the frequency, predictors and prevention of disengagement from ART care during pregnancy and postpartum are clearly required.

In conclusion, these results demonstrate that missed visits and disengagement from care occur frequently among HIV-positive women who initiate ART during pregnancy, particularly in the postpartum period. With the promotion of breastfeeding and a shift to lifelong ART for all pregnant HIV-positive women in SSA, these data highlight the importance of promoting postpartum retention and adherence both for PMTCT as well as for on-going maternal health. While additional research is required, women presenting for ANC or ART at later gestational ages may be particularly vulnerable and those newly diagnosed with HIV during pregnancy may require additional counselling and support to promote adherence and retention during pregnancy and after delivery.

## References

[1] Joint United Nations Programme on HIV/AIDS (2013). 2013 Progress Report on the Global Plan towards the elimination of new HIV infections among children by 2015 and keeping their mothers alive.

[2] Sturt A, Dokubo E, Sint T (2010). Antiretroviral therapy (ART) for treating HIV infection in ART-eligible pregnant women. Cochrane Database Syst Rev.

[3] Kumwenda J, Matchere F, Mataya R, Chen S, Mipando L, Li Q (2011). Coverage of highly active antiretroviral therapy among postpartum women in Malawi. Int J STD AIDS.

[4] National Department of Health (2013). The South African antiretroviral treatment guidelines.

[5] Ferguson L, Grant AD, Watson-Jones D, Kahawita T, Ong'ech JO, Ross DA (2012). Linking women who test HIV-positive in pregnancy-related services to long-term HIV care and treatment services: a systematic review. Trop Med Int Health.

[6] Myer L, Zulliger R, Black S, Pienaar D, Bekker L-G (2012). Pilot programme for the rapid initiation of antiretroviral therapy in pregnancy in Cape Town, South Africa. AIDS Care.

[7] Killam WP, Tambatamba BC, Chintu N, Rouse D, Stringer E, Bweupe M (2010). Antiretroviral therapy in antenatal care to increase treatment initiation in HIV-infected pregnant women: a stepped-wedge evaluation. AIDS.

[8] Stinson K, Jennings K, Myer L (2013). Integration of antiretroviral therapy services into antenatal care increases treatment initiation during pregnancy: a cohort study. PLoS One.

[9] Myer L, Zulliger R, Bekker L, Abrams E (2012). Systemic delays in the initiation of antiretroviral therapy during pregnancy do not improve outcomes of HIV-positive mothers: a cohort study. BMC Pregnancy Childbirth.

[10] Chibwesha C, Giganti M, Putta N, Chintu N, Mulindwa J, Benjamin J (2013). Optimal time on HAART for prevention of mother-to-child transmission of HIV. J Acquir Immune Defic Syndr.

[11] Van Schalkwyk M, Andersson MI, Zeier MD, La Grange M, Taljaard JJ, Theron GB (2013). The impact of revised PMTCT guidelines: a view from a public sector ARV clinic in Cape Town, South Africa. J Acquir Immune Defic Syndr.

[12] Denoeud-Ndam L, Fourcade C, Ogouyemi-Hounto A, Azon-Kouanou A, D'Almeida M, Azondékon A (2013). Predictive factors of plasma HIV suppression during pregnancy: a prospective cohort study in Benin. PLoS One.

[13] Fitzgerald FC, Bekker L-G, Kaplan R, Myer L, Lawn SD, Wood R (2010). Mother-to-child transmission of HIV in a community-based antiretroviral clinic in South Africa. S Afr Med J.

[14] World Health Organisation (2013). Consolidated guidelines on the use of antiretroviral drugs for treating and preventing HIV infection. Recommendations for a publich health approach.

[15] Nachega JB, Uthman O, Anderson J, Peltzer K, Wampold S, Cotton MF (2012). Adherence to antiretroviral therapy during and after pregnancy in low-income, middle-income, and high-income countries: a systematic review and meta-analysis. AIDS.

[16] Wang B, Losina E, Stark R, Munro A, Walensky RP, Wilke M (2011). Loss to follow-up in a community clinic in South Africa – roles of gender, pregnancy and CD4 count. South African Med J.

[17] MacPherson P, Lalloo DG, Choko AT, Mann GH, Squire SB, Mwale D (2012). Suboptimal patterns of provider initiated HIV testing and counselling, antiretroviral therapy eligibility assessment and referral in primary health clinic attendees in Blantyre, Malawi. Trop Med Int Health.

[18] Boyles TH, Wilkinson LS, Leisegang R, Maartens G (2011). Factors influencing retention in care after starting antiretroviral therapy in a rural South African programme. PLoS One.

[19] Kaplan R, Orrell C, Zwane E, Bekker L-G, Wood R (2008). Loss to follow-up and mortality among pregnant women referred to a community clinic for antiretroviral treatment. AIDS.

[20] Ngarina M, Popenoe R, Kilewo C, Biberfeld G, Ekstrom AM (2013). Reasons for poor adherence to antiretroviral therapy postnatally in HIV-1 infected women treated for their own health: experiences from the Mitra Plus study in Tanzania. BMC Publ Health.

[21] Watson-Jones D, Balira R, Ross DA, Weiss HA, Mabey D (2012). Missed opportunities: poor linkage into ongoing care for HIV-positive pregnant women in Mwanza, Tanzania. PLoS One.

[22] Stinson K, Myer L (2012). HIV-infected women's experiences of pregnancy and motherhood in Cape Town, South Africa. Vulnerable Child Youth Stud.

[23] Muchedzi A, Chandisarewa W, Keatinge J, Stranix-chibanda L, Woelk G, Mbizvo E (2010). Factors associated with access to HIV care and treatment in a prevention of mother to child transmission programme in urban Zimbabwe. J Int AIDS Soc.

[24] Bekker L, Myer L, Orrell C, Lawn S, Wood R (2006). Rapid scale-up of a community-based HIV treatment service. Programme performance over 3 consecutive years in Guguletu, South Africa. South African Med J.

[25] National Department of Health; South African National AIDS Council (2010). Clinical guidelines: PMTCT (Prevention of Mother-to-Child Transmission).

[26] Clouse K, Pettifor A, Shearer K, Maskew M, Bassett J, Larson B (2013). Loss to follow-up before and after delivery among women testing HIV positive during pregnancy in Johannesburg, South Africa. Trop Med Int Health.

[27] Tenthani L, Haas AD, Tweya H, Jahn A, van Oosterhout JJ, Chimbwandira F (2014). Retention in care under universal antiretroviral therapy for HIV-infected pregnant and breastfeeding women (‘Option B+’) in Malawi. AIDS.

[28] Ayuo P, Musick B, Liu H, Braitstein P, Nyandiko W, Otieno-Nyunya B (2013). Frequency and factors associated with adherence to and completion of combination antiretroviral therapy for prevention of mother to child transmission in western Kenya. J Int AIDS Soc.

[29] Black V, Hoffman RM, Sugar CA, Menon P, Venter F, Currier JS (2008). Safety and efficacy of initiating highly active antiretroviral therapy in an integrated antenatal and HIV clinic in Johannesburg, South Africa. J Acquir Immune Defic Syndr.

[30] Nassali M, Nakanjako D, Kyabayinze D, Beyeza J, Okoth A, Mutyaba T (2009). Access to HIV/AIDS care for mothers and children in sub-Saharan Africa: adherence to the postnatal PMTCT program. AIDS Care.

[31] Van Lettow M, Bedell R, Mayuni I, Mateyu G, Landes M, Chan AK (2014). Towards elimination of mother-to-child transmission of HIV: performance of different models of care for initiating lifelong antiretroviral therapy for pregnant women in Malawi (Option B+). J Int AIDS Soc.

[32] Stinson K, Myer L (2012). Barriers to initiating antiretroviral therapy during pregnancy: a qualitative study of women attending services in Cape Town, South Africa. African J AIDS Res.

[33] Otieno PA, Kohler PK, Bosire RK, Brown ER, Macharia SW, John-Stewart GC (2010). Determinants of failure to access care in mothers referred to HIV treatment programs in Nairobi, Kenya. AIDS Care.

[34] Boateng D, Kwapong GD, Agyei-Baffour P (2013). Knowledge, perception about antiretroviral therapy (ART) and prevention of mother-to-child-transmission (PMTCT) and adherence to ART among HIV positive women in the Ashanti Region, Ghana: a cross-sectional study. BMC Wom Health.

[35] Futterman D, Shea J, Besser M, Stafford S, Desmond K, Comulada WS (2010). Mamekhaya: a pilot study combining a cognitive-behavioral intervention and mentor mothers with PMTCT services in South Africa. AIDS Care.

[36] Rotheram-Borus M-J, Richter L, Van Rooyen H, van Heerden A, Tomlinson M, Stein A (2011). Project Masihambisane: a cluster randomised controlled trial with peer mentors to improve outcomes for pregnant mothers living with HIV. Trials.

[37] Kironde S, Mbule M, Kazibwe F Utilization of “mentor mothers” to support their peers for successful PMTCT service delivery: experiences from rural facilities with human resource limitations in East Central Uganda.

[38] Myer L, Harrison A (2003). Why do women seek antenatal care late? Perspectives from rural South Africa. J Midwifery Wom Health.

[39] Ebeigbe PN, Ndidi EP, Igberase GO, Oseremen IG (2010). Reasons given by pregnant women for late initiation of antenatal care in the niger delta, Nigeria. Ghana Med J.

[40] Lubega M, Musenze IA, Joshua G, Dhafa G, Badaza R, Bakwesegha CJ (2013). Sex inequality, high transport costs, and exposed clinic location: reasons for loss to follow-up of clients under prevention of mother-to-child HIV transmission in eastern Uganda – a qualitative study. Patient Prefer Adherence.

[41] Ekama SO, Herbertson EC, Addeh EJ, Gab-Okafor CV, Onwujekwe DI, Tayo F (2012). Pattern and determinants of antiretroviral drug adherence among Nigerian pregnant women. J Pregnancy.

[42] Mepham S, Zondi Z, Mbuyazi A, Mkhwanazi N, Newell ML (2011). Challenges in PMTCT antiretroviral adherence in northern KwaZulu-Natal, South Africa. AIDS Care.

[43] Vo BN, Cohen CR, Smith RM, Bukusi EA, Onono MA, Schwartz K (2012). Patient satisfaction with integrated HIV and antenatal care services in rural Kenya. AIDS Care.

[44] Suthar AB, Hoos D, Beqiri A, Lorenz-Dehne K, Duncombe C, McClure C (2013). Integrating antiretroviral therapy into antenatal care and maternal and child health settings: a systematic review and meta-analysis. Bull World Health Organ.

[45] Theuring S, Sewangi J, Nchimbi P, Harms G, Mbezi P (2014). The challenge of referring HIV-positive pregnant women with treatment indication from PMTCT to ART services: a retrospective follow-up study in Mbeya, Tanzania. AIDS Care.

[46] Stinson K, Boulle A, Coetzee D, Abrams EJ, Myer L (2010). Initiation of highly active antiretroviral therapy among pregnant women in Cape Town, South Africa. Trop Med Int Health.

[47] Kranzer K, Lewis JJ, Ford N, Zeinecker J, Orrell C, Lawn SD (2010). Treatment interruption in a primary care antiretroviral therapy programme in South Africa: cohort analysis of trends and risk factors. J Acquir Immune Defic Syndr.

[48] Geng EH, Glidden DV, Bwana MB, Musinguzi N, Emenyonu N (2011). Retention in care and connection to care among HIV-infected patients on antiretroviral therapy in Africa: estimation via a sampling-based approach. PLoS One.

[49] Holmes CB, Bengtson A, Sikazwe I, Bolton-Moore C, Mulenga LB, Musonda P Using the side door: non-linear patterns within the HIV treatment cascade in Zambia.

